# Stress Generation and Filament Turnover during Actin Ring Constriction

**DOI:** 10.1371/journal.pone.0000696

**Published:** 2007-08-08

**Authors:** Alexander Zumdieck, Karsten Kruse, Henrik Bringmann, Anthony A. Hyman, Frank Jülicher

**Affiliations:** 1 Max Planck Institute for the Physics of Complex Systems, Dresden, Germany; 2 Max Planck Institute for Molecular Cell Biology and Genetics, Dresden, Germany; Fred Hutchinson Cancer Research Center, United States of America

## Abstract

We present a physical analysis of the dynamics and mechanics of contractile actin rings. In particular, we analyze the dynamics of ring contraction during cytokinesis in the *Caenorhabditis elegans* embryo. We present a general analysis of force balances and material exchange and estimate the relevant parameter values. We show that on a microscopic level contractile stresses can result from both the action of motor proteins, which cross-link filaments, and from the polymerization and depolymerization of filaments in the presence of end-tracking cross-linkers.

## Introduction

During the division of eucaryotic cells, the cortical actin cytoskeleton plays a key role. At the end of mitosis, actin assembles at the site of cell division. As the assembly matures, a bundle of actin filaments and associated proteins is formed [1]. This bundle often forms a ring that encircles the cell. After maturation, the ring contracts and the cell is cleaved, a process called cytokinesis. The position of the cleavage furrow is determined by two signals induced by spindle microtubules [2]. It has been shown that forces are exerted at the cleavage furrow [3] suggesting that contractile stress in the bundle leads to normal forces in the membrane and consequently to the cleavage of the cell.

Cytokinesis is an active process that relies on the action of a number of proteins. Among these are myosin motor proteins that generate mechanical stress in the actin ring [4]. It has been suggested that sarcomere-like contractile elements play an important role in this process [5]. Other proteins involved in cytokinesis affect the nucleation, polymerization and depolymerization of actin filaments [1], [6]. These include capping proteins that stabilize polymerizing ends, formins that nucleate and polymerize actin filaments, the Arp2/3 complex that nucleates filament branches, and ADF/cofilin, which affects depolymerization. Finally, there are bundling and cross-linking proteins like α-actinin.

The kinetics of ring contraction has been observed in different cells. In *Schizosaccharomyces pombe* (fission yeast), the contraction velocity was found to be constant during cytokinesis [6]. In adherent *Dictyostelium* cells the observed contraction velocity decreased exponentially with time [7]. Myosin motors have been shown to play a key role for ring constriction [4]. The non-motor proteins also influence the contractile process. Measurements on fission yeast have shown that the velocity of ring contraction depends on factors that regulate the polymerization and nucleation of actin-filaments [6]. Remarkably, in fission yeast the turnover of actin and the associated proteins is rapid as compared to ring contraction [6].

It has been suggested that ring contraction can be described by a physical model that takes into account the balance of active contractile forces and hydrodynamic friction [7], [8]. In these calculations the contraction velocity was found to decrease with time. Computer simulations of such a model for the first division of sea urchin eggs that explicitly take into account flows of cytoplasmic material on the other hand lead to ring contraction with constant velocities [9].

Key aspects of cytokinesis can be discussed in a simplified geometry focusing on the properties of one-dimensional bundles forming contractile rings. The mechanical properties of filament bundles in the presence of motor proteins have been studied in vitro using purified systems. It has been shown that actin filament bundles in the presence of myosin motors contract spontaneously [10], [11]. Theoretical analysis of the relative filament sliding induced by active cross-linkers (e.g. motor aggregates) has revealed that contractile stresses can be generated even in bundles lacking a sarcomere structure [12]–[14]. In this case, motors and filaments self-organize to form a contractile filament configuration that is stable. Furthermore, as a result of motor action complex dynamic states can appear.

In this work, we present a multi-scale description of ring constriction. It consists of a microscopic model and a macroscopic phenomenological description, which are connected through an intermediate continuum description. Our phenomenological description is simple. Still, the processes we consider are sufficient to account for the observations on ring contraction in *C. elegans* and in fission yeast. In particular, we can predict from our analysis that the observed constant contraction velocity depends essentially on a sufficiently fast actin turnover. This result is not trivial, as is, for example, demonstrated by ring contraction in *Dictyostelium* [7]. There, the contraction velocity is not constant and processes distinct from the ones we consider have to be taken into account for describing the observed time course. The microscopic model incorporates an essential additional feature compared to previous models for bundle dynamics as it takes into account effects of filament assembly and disassembly, in particular, treadmilling. This extension is important for the dynamics of contractile rings for two reasons: first because our phenomenological analysis shows that a high actin turnover is essential for constant contraction velocities. Secondly, it allows for studying mechanisms of stress generation in contractile rings that do not rely on the action of force generation by myosins. Such mechanisms have so far not been addressed from a theoretical point of view.

## Results

### Ring constriction in the *C. elegans* embryo

**Figure 1:**
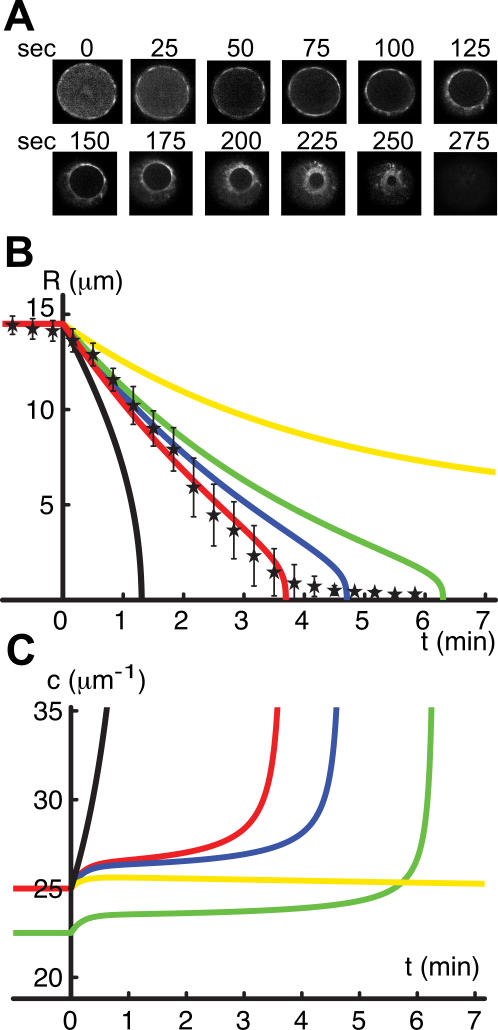
Dynamics of ring contraction. (A) Time lapse images of GFP tagged non-muscle myosin 2 during the first division using end-on imaging of a dividing C. elegans embryo at 21°C, which show the contracting ring structure. (B) Ring radius as a function of time. Stars indicate the average of eight experimentally observed ring radius traces as shown in (A). Bars mark one standard deviation. The colored lines represent solutions to Eqs. (1)–(4). For sufficiently large rate of filament turnover 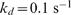, 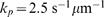, and using 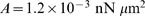, the calculated dynamics corresponds well to the observed one (red line). Reducing the polymerization rate to 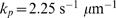, which implies a reduced initial filament concentration of 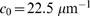 leads to slower ring contraction (green line). Using 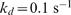 and decreasing the generated contractile stress using 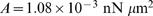 also leads to slower ring contraction (blue line). Using instead 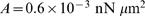 causes the ring to stop at a finite radius *R*≈5 µm (yellow line). In the absence of filament turnover, 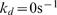 and with 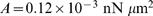, the contraction speed increases with time (black line). (C) Filament density c as a function of time shown for the same calculations as in (B). Parameter values are: initial ring radius *R*_0_ = 14.5 µm, initial filament density *c*_0_ = 25 µm^−1^, *N_b_* = 40, *K* = 10 nN µm^-1^, 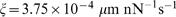.

We measured the ring diameter during the first cell division of a developing *C. elegans* embryo as a function of time, see Fig. 1A. The radius as a function of time averaged over eight such experiments is displayed in Fig. 1B (stars). Error bars indicate one standard deviation. The individual trajectories are displayed in the supporting text S1. The initial cell radius is R_0_≈14.5 µm and the ring contracts within 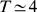 min. During most of the contraction process, the ring constricts with a constant speed of *v*_c_≈*R_0_/T*≈60 nm/s.

### Force balance and material exchange during ring contraction

**Figure 2:**
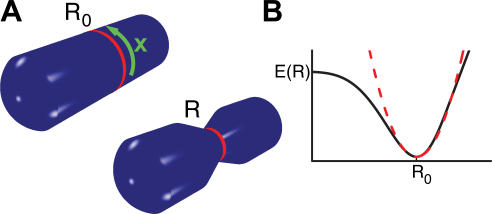
Illustration of key quantities used in the theoretical analysis. (A) Schematic representation of a cylindrical cell with a contractile ring of initial radius *R*_0_ (left) and contracted state with radius *R*<*R*_0_ (right). The position along the ring is denoted by *x*. (B) Schematic representation of the elastic energy *E(R)* as a function of ring radius (black). The dashed red line represents the approximation to *E(R)* given by Eq. (1), which we used in our calculations.

We now present a general theoretical analysis of the force and material balances during the contraction process. Using a simplified geometry, we consider a contractile ring wrapped around a cylindrical surface with radius R_0_ representing the cell, see Fig. 2A. The contractile stress ∑ in the ring is generated by active processes and will be specified below. It characterizes the mechanical work *δW* required to change the ring-diameter by *δR, δW = *2π∑*δR*, and has units of force. The contractile stress ∑ in the ring leads to a total force 2π∑ normal to the cylinder surface, which is balanced by viscous and elastic forces generated by the cell body. We describe the viscous forces associated with the cell constriction by 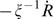, where 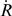 is the time derivative of the ring radius *R* and *ζ* ^−1^ an effective friction coefficient. The elastic response of the cell is described by the energy *E(R)*. The work required to induce a deformation is thus (∂*E*/∂*R*)*δ R*. A typical dependence of *E* on *R* is sketched in Fig. 2B (black line). We assume that the initial radius, *R = R_0_*, is locally stable. For simplicity, we approximate the elastic energy of cell deformations by
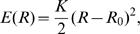
(1)where *K* is an elastic modulus of the cell (we have verified that different choices of *E(R)* do not change our results). Balancing the active forces of the ring with the viscous and elastic forces exerted by the cell leads to a dynamic equation for the ring radius
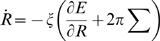
(2)The contractile stress ∑ depends on the internal dynamics of actin filaments and the associated proteins in the ring. Electron microscopy suggests that contractile rings consist of several distinct filament bundles [15]. Within each bundle, stress is generated by active processes, such as the action of motor protein aggregates, which link different filaments. Other possible contributions to active stress generation can result from filament polymerization in the presence of end-tracking cross-linkers. As will be shown below, the interaction of filament pairs leads to a quadratic dependence of the contractile stress on the filament density in a bundle. The total contractile stress in the ring can then be written as
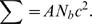
(3)Here, *A* is an effective material coefficient that characterizes force generation within a bundle and depends on the density of motors and other associated proteins, as well as on the rates of filament polymerization and depolymerization. We chose the sign of *A* so that positive stress is contractile. The number of distinct filament bundles in the ring is denoted *N_b_*, and *c* denotes the number density of filaments per unit length along a bundle and has units of inverse length. In general, the expression for ∑ will involve also other powers in *c*. In particular, for large densities the generated stress could be proportional to *c*. For small filament densities, however, the quadratic term should dominate and our main results are not affected by the specific choice of ∑.

A filament bundle of length *2πR* that consists of *N* filaments has a density *c = N/(2πR)*. Therefore, the filament density increases with decreasing radius in the absence of polymerization and depolymerization of filaments as *c*∼1/*R*. If we take into account filament turnover the filament density obeys the material balance equation
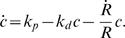
(4)Here *k_p_* and *k_d_* describe rates of filament assembly and disassembly. Note, that here assembly includes also nucleation of new filaments. For simplicity, we assume these parameters to be constant, which corresponds for example to a situation where the concentration of nucleators is constant. Equations (1)–(4) provide a physical description of the dynamics of ring contraction.

We solve these equations by numerical integration, starting at time *t* = 0 with *R* = *R_0_* and a steady state filament density c_0_ = *k_p_/k_d_*, which corresponds to an inactive ring with *A* = 0. Assuming that at *t* = 0 the ring is activated and starts to generate contractile stress according to Eq. (3), *A* is set to a positive value. The ring then starts to contract as described by Eq. (2). Figure 1B shows the ring diameter as a function of time for different parameter values. In the absence of filament turnover, the contraction velocity increases as a function of time because an increasing filament density leads to higher stress. A finite filament turnover time 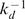 reduces this effect. If this time is short compared to the contraction time, the filament density is controlled by the assembly and disassembly kinetics of filaments, and 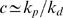 is roughly constant. In this case, the generated contractile stress ∑ remains constant. If contractile stresses dominate over elastic stresses, that is, if 2π∑>*KR_0_* as is required for robust contraction, the contraction velocity 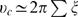 is constant during contraction, Fig. 1B (red line). For reduced contractile stress, contraction can slow down for small radii (blue and green) or even become incomplete (yellow). In the latter case the system reaches a steady state at finite radius at which contractile and elastic stresses balance. The corresponding filament densities are displayed in Fig. 1C.

### Comparison with experimental observations

Using appropriate parameter values, the calculated contraction dynamics corresponds well to the observed one see Fig. 1B (red line and stars). The ring in the *C. elegans* embryo contracts with constant velocity 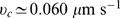. The parameters used in the calculation were *k_d_* = 0.1 s^−1^, *k_p_* = 2.5 s^−1^ µm^−1^, *N_b_* = 40, *A* = 1.2×10^3^ nNµm^2^, *ζ* = 3.75×10^−4^ µm nN^−1^s^−1^, *c*_0_ = 25 µm^−1^, *K* = 10 nNµm^−1^, and *R*_0_ = 14.5 µm.

We can relate these parameter values to structural, mechanical and kinetic properties of the actin cytoskeleton that have been observed in different systems. For filaments with a length 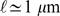, the turnover rate *k_d_* = 0.1 s^−1^ corresponds to a typical treadmilling velocity of 10 µm/min as is observed in reconstituted assays [16]. This value of *k_d_* is indeed fast compared to the ring contraction and thus ensures constant contraction velocity. The value of the polymerization rate *k_p_*_ =_ *k_d_c*_0_ is then fixed by the initial filament density. Our choice of *c*_0_ and *N_b_* is motivated by electron microscopy studies [15], [17].

The elastic modulus *K* is related to the whole cell elastic properties characterized by the Youngs modulus *Y* of the cell. A value of the order of 1 kPa has been found for dividing Ptk2 cells using AFM [18] and in the lamellipodium in fibroblasts using an optical stretcher [19]. We thus estimate the elastic constant of the cell 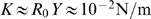. The contractile stress of the ring must exceed the elastic force, 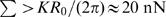. The stress in the contractile ring of echinoderm eggs has been measured by micromanipulation techniques. This suggest 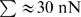 [20]. Using this value we find 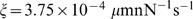 and *A* = 1.2×10^−3^ nNµm^2^.

A constant contraction velocity is also observed in fission yeast [6]. In this case the initial cell radius is R_0_≈1.9 µm_._. The total contraction time is 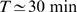. The fluorescent signal of GFP-Cdc4, a myosin light chain, does not change remarkably during contraction, which is consistent with a constant ring density. The fluorescence recovery of GFP-Cdc8 (tropomyosin) is shorter than 30s, which suggests a filament depolymerization rate *k_d_*≈0.04 s^−1^. Experiments suggest that only one bundle of about 20 filaments exists [1], [21], corresponding to *N_b_ = 1* and fixing *k_p_* = 0.8 µm^−1^ s^−1^. The contraction speed is 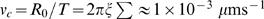. Assuming the same mechanical properties of an individual filament bundle as discussed above, we estimate Σ∼0.6 nN. From this estimate, we obtain 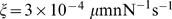. Note, that during cytokinesis in fission yeast a new cell wall is built while the ring contracts. We ignore effects of cell elasticity and assume 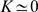. The dynamics that results from Eqs. (1)–(4) using these parameter values is consistent with experimentally observed contraction kinetics (see supporting text S1).

### Force generation in filament bundles by molecular motors

We now turn to the microscopic origin of stress generation in the contractile ring. Early experiments on sea urchin eggs have shown that myosin is involved in the generation of stresses in contractile rings [4]. As myosin minifilaments can displace actin filaments with respect to each other, a mechanism based on the sliding of actin filaments of opposite orientation has been proposed for stress generation in the contractile ring [3], [5]. Such a sliding filament mechanism is responsible for stress generation by sarcomeres in skeletal muscle cells [22].

**Figure 3:**
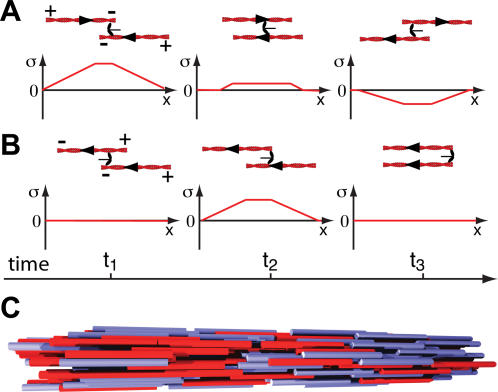
Stress generation in a filament bundle induced by plus end directed motor proteins. (A) Three stages of the relative sliding of two filaments of opposite orientation induced by a motor complex. During sliding, stresses result from the balance of motor forces, which are applied at the position where the motor is bound and friction forces that are distributed along the filaments. The combined stress profiles of both filaments σ(x) are displayed as a function of position *x*. When the centers of mass of the filaments slide towards each other (i.e. the pair contracts) the stress is positive (contractile) on average. When the filaments move apart, the stress is negative. Averaging over many filament pairs and motor position in the bundle, the net stress generated by this process is zero. (B) Filaments of equal orientation tend to align their plus ends if motors stay temporarily attached to the plus end. In this case, a positive (contractile) average stress is generated. Considering the combined effects of many filament pairs in a bundle, this process contributes to contractile bundle stress. (C) Snapshot of a stochastic simulation of a bundle consisting of many filaments that interact via the processes illustrated in (A) and (B). Displayed is a typical configuration in the steady state. Filaments oriented with their plus ends to the right and left are indicated by red and blue rods, respectively. This state has on average constant density as a function of position *x* and generates a net contractile stress along the bundle.

In vitro experiments show that bundles of myosin motors and actin filaments lacking sarcomeric structure can also contract [10], [11]. Theoretical analysis has revealed that contractile stresses can be generated in such bundles if interactions of filaments of the same orientation are taken into account [13], [14]. For the analysis it is assumed that motors act as mobile cross-linkers that temporarily link filament pairs and induce relative sliding between the filaments, see Fig. 3. Motor induced sliding of two filaments of opposite orientation will either shorten or lengthen the pair, depending on the relative filament position, see Fig. 3B. As a consequence, for a homogenous filament distribution, on average no contractile stress is generated in the bundle by this process. Sliding filaments of the same orientation, however, tend to shorten the filament pair. It can be shown that this process on average generates contractile stresses in the bundle [14] (see Fig. 3). Note that this implies that no organized sarcomere-like structure is required for the generation of contractile stresses in a bundle.

Motor-induced sliding of filaments of the same orientation occurs e.g. if an aggregate of plus-end directed motors that simultaneously binds to two filaments, reaches the end of one filament and stays attached for some time, see Fig. 3B. Other microscopic mechanisms can amplify these effects [23].

We illustrate this behavior by a simple stochastic model for the dynamics of filaments along a common bundle axis. In this model, filaments can interact if they overlap. In a time-step of duration Δt pairs of overlapping filaments are randomly selected and displaced relative to each other in a direction, which depends on their relative orientation and a distance that depends on the interaction strength, see Fig. 3 (see supporting text S1 for details). Finally, we impose periodic boundary conditions, where the system length *L* = 2π*R* is the ring circumference and assume that all filaments are of the same length *l*.

We start in our simulations with an initially homogenous distribution of filaments. Depending on the value of the interaction strength of parallel filaments α̅, which has units of inverse time, the system reaches different dynamic states after long times. If α̅ is smaller than a critical value α̅*_c_*, the filament distribution remains homogenous. In this case, a contractile stress is generated in the bundle (see supporting text S1 for details). For α̅>α̅*_c_*, the initially homogeneous bundle is unstable and develops into an inhomogenous distribution that is either a stationary distribution of segregated filaments or displays complex dynamics such as propagating density profiles. The critical value α̅*_c_* is positive and depends on the interaction strength β̅ of filaments of opposite orientation.

### Force generation in filament bundles by filament polymerization and depolymerization

**Figure 4:**
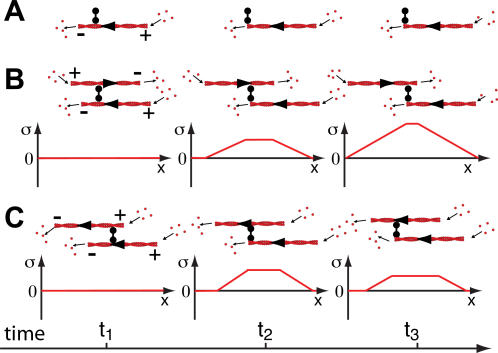
Stress generation in a bundle of treadmilling filaments and passive cross-linkers in the absence of motor proteins. (A) Three stages of the treadmiling of a pair of anti-parallel filaments. The arrows at the filament ends indicate that monomers are added at the plus ends and removed from the minus ends. As a consequence of treadmilling, in the absence of cross-linkers, the centers of mass of the filaments move relative to each other. At the same time, fixed subunits along the filaments do not move. Stresses can be generated if filaments are linked by passive cross-linkers, which have the ability to bind to filaments and also stay attached to depolymerizing filament ends (end-tracking cross-linkers). No stress is generated if monomers are cross-linked along filaments since these monomers do not move (left). As soon as one depolymerizing filament end is linked to the second filament by a cross-linker (middle), both filaments are physically moved relative to each other by depolymerization forces. This leads to a stress profile along the filament pair with positive (contractile) stress (middle and right). This process therefore contributes to contractile average bundle stress if many filament pairs are interacting in a filament bundle. Note that this is different from stress generation by motors, where anti-parallel filaments do not contribute to stress. (B) Stress profiles for a pair of treadmiling filaments that are arranged in parallel. Again, if a depolymerizing end of one filament is linked to the second filament, relative sliding occurs which is driven by filament depolymerization. The resulting stress is positive (contractile).

The polymerization and depolymerization of filaments can also generate forces [24], [25]. In a filament bundle this can contribute to stress generation if end-tracking cross-linker are present [26]. Such proteins can bind along filaments and stay bound to depolymerizing filament ends for some time, Fig. 4A. Such forces might be important for cytokinesis in particular in the absence of myosin II motors [27]. We can extend our description to include filament treadmilling and the role of end-tracking cross-linkers. Treadmilling leads to spontaneous motion of filaments with respect to the surrounding fluid, even in the absence of interactions with other filaments. In the presence of end-tracking cross-linkers, treadmilling can induce relative sliding between filaments of the same and opposite orientation, see Fig. 4. Simulating this dynamics analogously to the case of motor-induced filament sliding, we find again cases of stable homogenous bundles and cases where homogeneous bundles are unstable and complex dynamics emerges. The critical value of the interaction strength *α̅*′ mediated by end-tracking cross-linkers depends on the treadmilling velocity *v* and the interaction strength of filaments of opposite orientation. Remarkably, even in a homogenous bundle the interaction between anti-parallel filaments now leads to a net contractile stress, see Fig. 4.

### Continuum description

The description of force and stress generation in filament bundles is stochastic in nature and based on microscopic interactions. In order to find simple expressions for the average stress and to connect the microscopic description with the phenomenological description of ring contraction, we use a coarse-grained continuum description, which can connect the different scales. By characterizing the system via the densities *c* ^±^(x) of filaments pointing with their plus end in the positive and negative *x*-direction, respectively, we obtain a deterministic continuum description for the dynamics, which is given by equations of the form
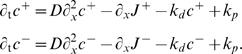
Average sliding of filaments induced by active processes is described by the currents *J* ^±^. The stochastic component of the same processes leads to diffusive motion characterized by an effective diffusion coefficient D. The currents can be expressed in terms of the densities. Interactions of filament pairs correspond to currents, which are quadratic in densities. For the case of motor induced sliding, these currents have been presented in [13], [14]. In the case of filament sliding induced by end-tracking cross-linkers, the currents can be decomposed as 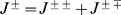, where
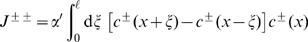
describes interactions between filaments of the same orientation and
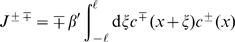
interactions between filaments of opposite orientation. Here, α and β are effective interaction strengths with units of velocity. They are related to the rates α′ and β′ of the stochastic model by a coarse graining procedure. Similarly, α and β denote the corresponding interaction strengths for motor induced filament sliding. These coefficients capture the effective strength of the respective interactions including protein concentration as well as microscopic details of the interaction.

In this continuum description we find stable homogeneous density profiles as well as profiles with propagating and oscillating density distribution. In the case of filament treadmilling, we also find extended regions with stationary filament patterns. We can furthermore calculate coarse-grained profiles of the average stress in the bundle [14]. In the special case of a homogeneous bundle, we find
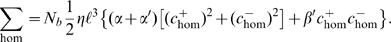
Here, *n* denotes an effective viscosity characterizing the dynamics of filaments in the bundle and *l* is the filament length. Note, that on average interactions induced by motors (interaction strength α) and by end-tracking cross-linkers (strength α′) contribute to contractile bundle stress if filaments have the same orientation. For filaments pointing in opposite directions, end-tracking cross-linkers generate a contribution to stress proportional to β′, while motors do not contribute in this situation. This is due to the symmetry of this interaction that leads to positive (contractile) as well as negative (expansive) stresses in the filament pair, cf. Fig. 3A. Note, that while the stress depends explicitly on the filament length *l*, its dependence on the myosin concentration is implicitly contained in the parameters α, α′, β, and β′.

## Discussion

In summary, we presented a phenomenological description of the dynamics of a contractile filament ring. Our study is motivated by the constriction of a contractile ring in eucaryotic cells during cytokinesis. In our description, the ring dynamics is driven by the contractile stress in the ring and accounts for effects of filament turnover. Furthermore, we have discussed physical mechanisms of stress generation by active processes and have shown that in addition to motor proteins the depolymerization of filaments also can contribute to contractile stress in the presence of end-tracking cross-linkers.

We compared our results to the observed ring constriction during the first division of the *C. elegans* embryo. Our calculations can account for the observed contraction with constant velocity if filament turnover is sufficiently fast. A constant contraction velocity is also observed in fission yeast. There, the filament turnover rate is also known and sufficiently fast to lead to constant filament density as required for constant velocity.

We can relate our phenomenological description to more microscopic models of force generation. Our minimal model is based on the pair wise interaction of filaments and is valid if the density of cross-linkers is low. This assumption is most likely not satisfied in the cleavage furrow, where a high density of active elements is able to generate large forces of the order of tens of nanoNewtons [20]. In a situation of high cross-linker density, individual filaments experience larger forces since they are transiently linked to clusters of other filaments via active and passive cross-linkers and the generated contractile stresses can increase. This more complex situation is not captured by our simple description. However, in the context of our model, such a situation corresponds to a case where individual filaments feel effective friction forces in the cross-linked filament network. Temporary cross-links keep filaments together only for a certain time. In the presence of forces, filaments will still slide with respect to each other but with significantly reduced velocity. This corresponds to an increase of the viscosity *η* in our description, which now becomes an effective viscosity that takes friction forces resulting from cross-linking into account. By choosing an appropriate effective viscosity in the bundle, which exceeds the viscosity of the solvent we can describe the contractile stress values relevant to the cleavage furrow. Note that this friction is different from the friction that characterizes ring contraction introduced in the phenomenological description. This latter friction depends on the viscosity of the cytoplasm and cortical protein networks that are formed during ring contraction.

In our minimal model, the contractile bundle stress is generated both by motor proteins as well as by filament depolymerization together with end-tracking cross-linkers. From our comparison to experiments, we can estimate the material parameter *A* that describes stress generation in the bundle and can be linked to the parameters of the minimal model by 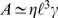. Here, *n* is the effective bundle viscosity, *l* denotes the typical filament length, and γ is an effective velocity of relative filament sliding, which can be related to the interaction strengths of the active processes as 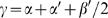. Using *γ* = 1 µm/s, I≈1 µm, and *A*≈1.2×10^−12^Nµm^2^ estimated for the *C. elegans* embryo, we find *η*≈1.2 Nsm^−2^. This corresponds to about 1200 times the viscosity of water. To describe filament bundles that are highly crosslinked and where large numbers of filaments interact simultaneously by active processes remains an important challenge. On a more coarse-grained level, the concept of active gels can provide a general description of the physics of highly cross-linked cytoskeletal systems [28]-[30].

## Materials and Methods

### End-on imaging of *C. elegans*

The worms were cultured as described [31]. NMY2::GFP [32] worms were maintained at 16° C and shifted to 25° C for 24h before an experiment. Then the worms were mounted on slides, covered with cell tac (BD Bioscience) in 10mM Tris Cl, pH 8.5. The embryos were put on their ends using a micromanipulator-controlled glass needle. Embryos were filmed using spinning disk microscopy at 23° C as described [2].

## Supporting Information

Supporting Text S1 (0.91 MB PDF)
