# Supplementary material for: Stress Generation and Filament Turnover during Actin Ring Constriction
Source: PLoS One. 2007 Aug 8;2(8):e696. doi: 10.1371/journal.pone.0000696 (PMC1936222; doi:10.1371/journal.pone.0000696)
Supplement: Supporting Text S1 — (0.91 MB PDF) [file pone.0000696.s001.pdf]

Supporting Material: Stress Generation and Filament Turnover During Actin Ring Constriction

Alexander Zumdieck[1,†], Karsten Kruse[1,*], Henrik Bringmann[2], Anthony A. Hyman[2], and Frank Jülicher[1,3]
[1]Max Planck Institute for the Physics of Complex Systems, 01187 Dresden, Germany
[2]Max Planck Institute for Molecular Cell Biology and Genetics, 01307 Dresden, Germany
[3] Corresponding author.
[†] present address: Physicochimie Curie (CNRS-UMR 168), Institut Curie,
Section de Recherche, 26 rue d'Ulm, 75248 Paris Cedex 05, France
[*] present address: Theoretische Physik, Universität des Saarlandes, 66041 Saarbrücken, Germany
(Dated: April 20, 2007)

A. Supplemental Data on *C. elegans* Ring Contraction

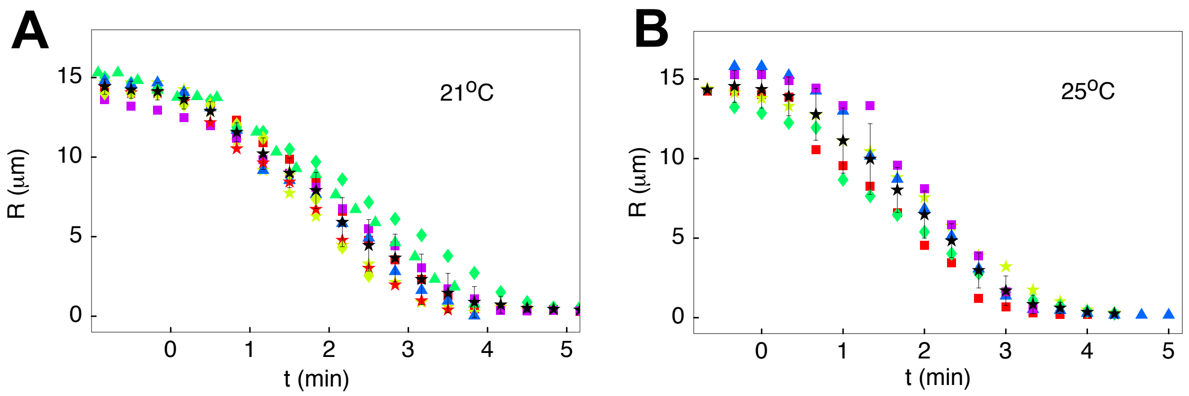

FIG. S1: Contracting rings in *C. elegans* embryos. (A) Traces of eight different embryos at 21° C and their average (stars) with standard deviation. (B) Traces of five embryos at 25° C and their average (stars).

The contraction dynamics in *C. elegans* embryos are very similar between different embryos but depend on parameters such as temperature. Figure S1 shows the traces for eight embryos at 21° C and five embryos at 25° C. Note that the contraction occurs faster at 25° C. The constant contraction velocity is $4.7 \pm 0.09\,\mu$m/minute at 25° C and $3.7 \pm 0.1\,\mu$m/minute at 21° C.

B. Numerical Simulation of Active Filament Bundles

In this section we provide details on the simulation of active bundles of interacting filaments. We study the behavior of N individual filaments of length ℓ along a linear position coordinate x. Corresponding to a ring with circumference L, we impose periodic boundary conditions with period L. The position of the center of filament i with $i = 1, .., N$ is denoted by x_i. Its orientation with respect to the positive x-axis is denoted by σ_i. This orientation is $\sigma_i = +1$ if the plus end points in positive x-direction, $\sigma_i = -1$ implies that the minus end points in x-direction. At every timestep of duration Δt, each filament is displaced by a distance Δ_i as a result of diffusion, treadmiling, and the interaction with molecular motors as well as with passive cross-linkers. Furthermore, filaments are removed and created to capture effects of polymerization and depolymerization of filaments. We assume that filaments cannot change their orientation.

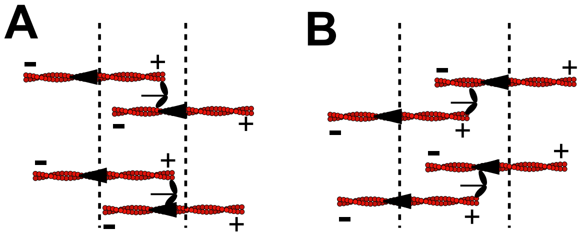

FIG. S2: The direction of filament sliding depends on the relative position of the two parallel filaments. The vertical lines mark the initial positions of the filament centers (top). After the action of the molecular motors the filaments have shifted (bottom). Both filaments in a pair move in mutually opposite directions due to conservation of momentum. (a) The top filament is located left of the other filament and moves to the right. (b) The top filament is located right of the other and moves to the left.

1. *Stochastic Simulation*

Diffusion is described by a random displacement $\Delta_i^{(D)}$ with zero average and Gaussian distribution with variance $\bar{D}\Delta t$:

$$P(\Delta_i^{(D)}) = \frac{1}{\sqrt{2\pi \bar{D}\Delta t}} \exp(-\frac{{\Delta_i^{(D)}}^2}{2\bar{D}\Delta t}) \quad , \tag{S1}$$

where the parameter $\bar{D}$ is the effective diffusion coefficient of filaments of length ℓ.

Treadmilling of filaments is characterized by the treadmilling speed $\bar{v}$. Per time step Δt the filaments are displaced by

$$\Delta_i^{(v)} = \sigma_i \bar{v} \Delta t \quad . \tag{S2}$$

Note that the direction of displacement depends on the filament orientation σ_i.

If two filaments overlap, i.e. $|x_i - x_j| < \ell$, they may interact via molecular motors or passive crosslinkers. This is captured by determining stochastically for each filament if it interacts with another filament during a given time step.

The interaction between parallel filaments due to motor dimers is described as follows: For each filament i we count all candidates ($n_i^{(c,\alpha)}$) for interaction. Candidates are filaments that have the same orientation and overlap with i. The probability of an interaction between filament i and a candidate filament to take place during the time interval Δt is then given by

$$p_i^{(\alpha)} = \bar{\alpha} n_i^{(c,\alpha)} \Delta t \in [0; 1] \quad . \tag{S3}$$

Here $\bar{\alpha} \in [0; (N\Delta t)^{-1}]$ is the rate of interactions between parallel filaments due to molecular motors in the stochastic simulation. If $p_i^{(\alpha)}$ is larger than a random number drawn from a uniform distribution on $[0; 1]$, a filament k is chosen at random from the candidate filaments. The displacements for the two parallel filaments i and k then depend on their relative positions (cf. Fig S2):

$$\Delta_i^{(\alpha)} = \frac{\delta}{2}, \; \Delta_k^{(\alpha)} = -\frac{\delta}{2} \;\; \text{for} \;\; x_i < x_k \tag{S4}$$

$$\Delta_i^{(\alpha)} = -\frac{\delta}{2}, \; \Delta_k^{(\alpha)} = \frac{\delta}{2} \;\; \text{for} \;\; x_i > x_k \tag{S5}$$

respecting the conservation of momentum of the filament pair. Here we have assumed that during the time Δt only one motor executes a power stroke, so that δ is the working distance of one motor head [1]. Multiple powerstrokes during a time step Δt can be captured by using multiples of δ in Eqs. (S4) and (S5).

The interaction between anti-parallel filaments due to molecular motors is very similar. In this case filaments have to be of opposite orientations and the probability for an interaction to occur is given by

$$p_i^{(\beta)} = \bar{\beta} n_i^{(c,\beta)} \Delta t \in [0; 1] \quad , \tag{S6}$$

where $\bar{\beta} \in [0; (N\Delta t)^{-1}]$ is the rate of interactions between anti-parallel filaments due to molecular motors in the stochastic simulation similar to $\bar{\alpha}$ in Eq. (S3). The change in filament position however now depends on filament orientation but is independent of the relative position:

$$\Delta_i^{(\beta)} = \frac{\delta}{2},\ \Delta_k^{(\beta)} = -\frac{\delta}{2} \ \text{ for } \ \sigma_k = 1 \tag{S7}$$

$$\Delta_i^{(\beta)} = -\frac{\delta}{2},\ \Delta_k^{(\beta)} = \frac{\delta}{2} \ \text{ for } \ \sigma_k = -1 \quad . \tag{S8}$$

Passive cross-linkers are described by similar procedures. The dynamics for parallel filaments is identical for motors or passive cross-linkers, so that the stochastic procedures for cross-linkers and motors between parallel filaments are the same. The only difference is that passive cross-linker interaction occurs with rate $\bar{\alpha}'$ and that filaments are moved by the length of a monomer a instead of the working distance δ. For anti-parallel filaments the interaction occurs with rate $\bar{\beta}'$. The dynamics is however different from that induced by motors. As a passive cross-linker is only effective when attached to a minus end, the range of positions of a candidate filament j then depends on the orientation of the reference filament σ_i:

$$0 < x_i - x_j < l \ \text{ for } \ \sigma_i = 1 \tag{S9}$$

$$0 < -(x_i - x_j) < l \ \text{ for } \ \sigma_i = -1 \quad . \tag{S10}$$

In this position range, only filaments of the opposite orientation are candidates, $\sigma_i = -\sigma_j$.

The filament displacements are similar to those in Eqs. (S7) and (S8). Here we replace the distance δ by the monomer length a:

$$\Delta_i^{(\beta')} = \frac{a}{2},\ \Delta_k^{(\beta')} = -\frac{a}{2} \ \text{ for } \ \sigma_k = 1 \tag{S11}$$

$$\Delta_i^{(\beta')} = -\frac{a}{2},\ \Delta_k^{(\beta')} = \frac{a}{2} \ \text{ for } \ \sigma_k = -1 \quad . \tag{S12}$$

The new position at time $t + \Delta t$ is determined by

$$\begin{aligned} x_i(t + \Delta t) \ &= \ x_i(t) + \Delta_i^{(D)} + \Delta_i^{(v)} \\ &\quad + \Delta_i^{(\alpha)} + \Delta_i^{(\beta)} + \Delta_i^{(\alpha')} + \Delta_i^{(\beta')} \end{aligned} \tag{S13}$$

Besides being displaced, individual filaments are depolymerized with rate $\bar{k}_d$ and simply vanish. During each time step up to N_0 filaments are generated with probability $\bar{k}_p \Delta t$. Their position and orientation are random. The steady state number of filaments is given by $N_0 = \bar{k}_p / \bar{k}_d$.

Parameters used in the simulations are $L = 5$, $\delta = 0.005$, $a = (1/370)$, $\bar{\alpha}\Delta t = 40$, $\bar{\beta}\Delta t = 40$, $\bar{\alpha}'\Delta t = 40$, $\bar{\beta}'\Delta t = 40$, $v\Delta t/\ell = 0.5$, $\bar{k}_p \Delta t = 0$, $\bar{k}_d \Delta t = 0$, unless stated otherwise. Lengths are measured in units of the filament length ℓ. A typical value for the distance covered by one powerstroke of a myosin motor is $\delta = 5\,\text{nm}$ [1–3]. One μm of actin filament contains 370 monomers [4]. For filaments of length $\ell = 1\,\mu$m the values for δ and a thus reflect the ratios $\ell/\delta = 200$ and $\ell/a = 370$ as measured for myosin [1] and actin [4] respectively.

C. Stress

A motor complex that exerts a force on two filaments in a pair induces stress in each of the filaments. Assuming that the stress vanishes at the free filament ends and varies linearly along the filament we obtain profiles such as sketched in Figs. 3 and 4 of the main text. We choose the signs so that pulling towards the motor position induces a positive stress. The contractile stress along a filament with its center of mass located at x_1 due to a motor bound at position x_m is given by

$$\sigma_1(x) = \begin{cases} (f_m/\ell)(x - x_1 + \ell/2) & \text{for } \ x_1 - \ell/2 \le x \le x_m \\ (f_m/\ell)(x - x_1 + \ell/2) - f_m & \text{for } \ x_m \le x \le x_1 + \ell/2 \end{cases} \tag{S14}$$

and for the filament located at x_2 analogously. The resulting contractile stress profile for the entire filament pair is then given by

$$\sigma(x) = \begin{cases} (f_m/\ell)(x - x_1 + \ell/2) & \text{for } \ x_1 - \ell/2 \le x \le x_2 - \ell/2 \\ (f_m/\ell)(x_2 - x_1) & \text{for } \ x_2 - \ell/2 \le x \le x_1 + \ell/2 \\ -(f_m/\ell)(x - x_2 - \ell/2) & \text{for } \ x_1 + \ell/2 \le x \le x_2 + \ell/2 \end{cases} \quad . \tag{S15}$$

The contractile stress $\Sigma(x)$ in a bundle of interacting filament pairs can then be computed by adding up all contributions from individual pairs at all positions x.

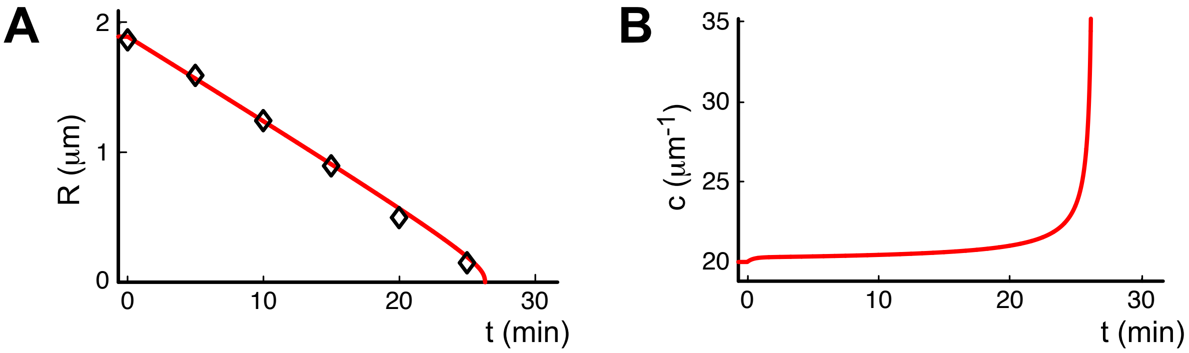

FIG. S3: Radius and filament density of a contracting ring as a function of time obtained by numerical integration of Eqs. (1)-(4) . (a) Ring radius R as a function of time t. The red line agrees with experimentally obtained data for wildtype fission yeast indicated by diamonds (from [5]). Note that fission yeast cells are smaller than *C. elegans* and that ring constriction is slower. Parameters are: $R_0 = 1.9\,\mu$m, $k_d = 0.04\,\mathrm{s}^{-1}$, $c_0 = 20\,\mu\mathrm{m}^{-1}$, $k_p = 0.8\,\mathrm{s}^{-1}\mu\mathrm{m}^{-1}$, $N_b = 1$, $\xi = 3 \times 10^{-4}\,\mu\mathrm{mnN}^{-1}\mathrm{s}^{-1}$, and $A = 1.4 \times 10^{-3}\,\mathrm{nN}\mu\mathrm{m}^2$. The corresponding mechanical, contractile stress is $\Sigma \simeq 0.56\,$nN. The cell elastic modulus is chosen to be $K = 0$, as we ignore elastic effects. (b) Filament density c as a function of time.

D. Fission Yeast Dynamics

Our theoretical description of ring contraction as given by Eqs. (1)-(4) can also be applied to yeast. In fission yeast (*S. pombe*) the contractile ring also constricts with constant velocity [5], see diamonds in Fig. S3. Fission yeast is smaller than *C. elegans* ($R_0 = 1.9\,\mu$m), possesses a more rigid outer shell and during ring constriction a new cell wall is formed in the middle of the cell. This suggests that the deformation of the outer cell wall might not be necessary for cytokinesis. We ignore elastic effects and set $K = 0$. Ring contraction in yeast is slow with $T \simeq 30\,$min and $v_c \simeq 1\,\mathrm{nm\,s}^{-1}$. In cells with reduced actin dynamics, the observed contraction velocity is smaller than in wild type [5] (data not shown). In our description a reduced contraction speed results also when filament turnover is reduced. The reason for this effect is that reduced filament turnover implies a reduced contractile stress Σ due to a less effective contribution of polymerization forces to stress generation in the ring.

[1] Howard J (2001) Mechanics of Motor Proteins and the Cytoskeleton. Sunderland, MA: Sinauer Press.
[2] Veigel C, Coluccio L, Jontes J, Sparrow J, Milligan R, et al. (1999) The motor protein myosin-I produces its working stroke in two steps. Nature 398:530–3.
[3] Veigel C, Bartoo M, White D, Sparrow J, Molloy J (1998) The stiffness of rabbit skeletal actomyosin cross-bridges determined with an optical tweezers transducer. Biophys J 75:1424–38.
[4] Pollard T, Borisy G (2003) Cellular motility driven by assembly and disassembly of actin filaments. Cell 112:453–65.
[5] Pelham R, Chang F (2002) Actin dynamics in the contractile ring during cytokinesis in fission yeast. Nature 419:82–6.
